# On the Nature and Sources of the Contents of the Fœtal Stomach, Being the Substance of a Paper Communicated to the Royal Society of London, in June Last

**Published:** 1847-03

**Authors:** George Robinson

**Affiliations:** Fellow of the Royal Medical and Chirurgical Society of London, and Joint Lecturer on Materia Medica and Forensic Medicine in the Newcastle-on Tyne Medical School


*On the Nature and Sources of the Contents of the Fœtal Stomach, being the substance of a Paper communicated to the Royal Society of London, in June last.* By GEORGE ROBINSON, M. D., Fellow of the Royal Medical and Chirurgical Society of London, and Joint Lecturer on Materia Medica and Forensic Medicine in the Newcastle-on Tyne Medical School.—Whilst all physiologists who have examined the appearances presented by the alimentary canal of the fœtus, agree in representing *the small intestines* as actively engaged in the function of digestion, a remarkable difference of opinion has prevailed as to the source of the nutritious matter there submitted to that process. Harvey, who, of modern physiologists, alone supposes it to enter the intestine through the stomach, adopts the views of the older writers, and concludes, from his observations, that it is the liquor amnii swallowed by the fœtus, which affords the material for chylification. Geoffroy Saint-Hilaire, perceiving the anatomical objection to this doctrine, which arises from the fact of similar appearances have been found in the intestinal canal of fœtuses born with an imperforate œsophagus, would seem to suppose that the superior portion of the intestines, being irritated by its contact with the bile, secretes a nutritive mucus, by the digestion of which chyle is formed. And Dr. Robert Lee, who is, I believe, the most recent investigator of this subject, has been led by his researches to the conclusion that the liver is the source of the nutritious fluid found in the alimentary canal of the fœtus; the function of that gland being, in his opinion, not merely that of separating from the blood an excrementitious substance, but also that of pouring into the fœtal intestines, through the hepatic duct, a quantity of albuminous fluid.

Now, even though a quantity of albumen may be present in the bile taken from the hepatic duct, it is surely desirable that the impossibility of the nutritive contents of the small intestine having been derived from the fœtal stomach should be clearly demonstrated, before we admit the correctness of this latter conclusion as to their source. But it will be seen by a reference both to systematic writers, and to the authors who have more expressedly treated of the fœtal functions, that the evidence yet advanced is by no means sufficient to establish any positive opinion on this point.

Dr. Lee thus describes the result of his own examination of the stomach in several human fœtuses: "The stomach of the fœtus I usually found in these cases distended with a semi-transparent, ropy, mucous, and occasionally acescent fluid without any sensible admixture of albuminous or other apparently nutritious matter." Dr. Blundell, whose opinions on any point connected with the uterine or fœtal functions I consider to be worthy of every attention, has but the following brief allusion to the subject: "The stomach of the fœtus is not unfrequently empty, or nearly so ; and when it does contain anything, if I may judge from some two or three observations, this matter consists of a mucous secretion mixed with the gastric juice." And in the systematic works on physiology, I do not perceive any mention of the subject further than an incidental statement that the fœtal stomach contains a mucous fluid. The time which I could myself devote to this inquiry, and my opportunities for conducting it, have been too limited to enable me to attempt anything like a full and final settlement of the various questions connected with this department of fœtal physiology. The few facts which have fallen under my notice are, however, sufficiently distinct to establish some positive inferences, and I can only hope that the results of this rude effort may induce others better qualified for the investigation to extend and complete our knowledge of this interesting group of functions.

My attention was first attracted to the subject in the autumn of last year, by an accidental observation of the stomach of a fœtal rabbit, and having subsequently examined the fœtuses of other animals, I met with a number of curious facts, of which the following is a brief account.

*Observation* 1. During the last ten days of its uterine existence, the stomach of the fœtal rabbit is invariably distended with a semi-transparent fluid, of a dark green colour, extremely viscid, and coagulating by heat into a solid opaque mass. Viewed microscopically, it is found to consist of a clear liquid, suspended in which are numerous cells of different shapes, and several large globules of oil. When heated in a watch glass over a spirit lamp, it assumes the appearance of a piece of baked custard ; and on dropping it into boiling water, it also instantly solidifies. Dried by a more gentle heat, it forms a brittle substance like gum, which, on being dissolved in water, answers to every test of albumen. A small quantity of the fresh liquid which had been excluded from the air in a test tube, on being examined at the end of six months, though it had acquired a disagreeable odour, was found to retain all its chemical properties, coagulating as readily as when recently obtained. The stomach was in these cases extremely pale, and presented very little vascularity; whereas the small intestines were plentifully supplied with blood-vessels. The chymous substance which the latter contained, was evidently derived from the stomach, becoming however more opaque, as it gradually assumed the situation of the meconium. This latter substance, of a bright green colour, exhibited a curious reaction with strong nitric acid, the addition of a few drops of the latter instantly causing it to assume a bright scarlet hue.

In three instances, a liquid, precisely resembling that contained in the stomach, escaped from the mouth of the animal as it lay on its side before being opened; and in two other cases, the same substance was found in the œsophagus. The liquor amnii of these animals is a transparent, almost colourless fluid, exhibiting scarcely any indication of the presence of albumen. In two instances where I had an opportunity of watching the fœtus through the transparent membranes, it was observed to swallow the liquor amnii, and from its continuing to perform the act of deglutition after its removal from the uterus, the stomach was found extremely distended with air, each mouthful of which appearing as a little vesicle, was preserved distinct by the viscidity of the stomachic fluid. The facts detailed in this observation are drawn from the examination of more than thirty animals.

*Observation* 2. The uterus of a rabbit, killed about the *tenth day* after impregnation, contained six fœtuses, an inch and a half in length, and the tissues of which were still very gelatinous. Through the transparent walls of the abdomen, the dark globular stomach of each was distinctly visible, and on opening the peritoneal cavity, it constituted, with the exception of the liver, the most prominent object presented to the eye, being in every case distended with a liquid of a dark green colour. This fluid was perfectly transparent, *presented no microscopical object, was not at all viscid, and did not undergo any change on the application of heat or nitric acid.*

The intestines were in these animals extremely minute and tortuous, translucent, and almost void of meconium, or any other coloured substance. The quantity of liquor amnii was greater than that met with in the former observations, and its opacity, on the application of the tests for albumen, if anything rather more distinct.

*Observation* 3. In a fœtal lamb examined at a very early period (for its weight did not exceed two ounces,) the stomach contained two drachms of a clear, citron-coloured liquid, which was neither viscid, nor did it present any opacity or change on the application of heat and nitric acid. The quantity of liquor amnii was relatively large; six ounces of it were collected, and in its colour, consistence, and chemical properties, it precisely resembled the fluid obtained from the fœtal stomach. The intestines were very minute and tortuous, and with the exception of a small quantity of serous fluid, apparently empty.

*Observation* 4. In another fœtal lamb which was fully developed and ready for birth, the stomach, on being opened, presented a substance differing very much from the liquid just described. It contained an ounce of a viscid, transparent semifluid mass, suspended in which and gradually subsiding in it, so as to form a sediment, were numerous minute, oval, brown particles, in shape and colour very much resembling grains of linseed, but somewhat larger, and around each of them was a quantity of an extremely tenacious, gelatinous substance of a yellow colour. Neither the supernatant liquid nor the liquor amnii evinced the presence of albumen, though both possessed considerable viscidity. A substance precisely similar to the yellow gelatinous sediment found within the stomach, existed in considerable quantities upon the fleece, the legs, and the whole exterior of the animal; *but on the most careful examination I could not detect in any other part of the fœtal structures or appendages, a single particle corresponding to the brown oval masses met with in the stomach.*

The intestines, in addition to some meconium, contained a chymous mass, the liquid portion of which was distinctly albuminous.

*Observation* 5. The liquor amnii of a fœtal calf, (which was examined at a very early period, when its body weighed only nine ounces) was of a citron colour, neither viscid nor coagulable by heat or nitric acid, but instantly formed an opaque membranous coagulum on the addition of a little acetate of lead, or proto-nitrate of mercury. The stomach of the same animal contained two drachms of a fluid, which in every respect resembled the liquor amnii, and which, on standing, did not present any sediment or coloured flocculi. In the intestine was a small quantity of meconium, which, on being heated with nitric acid, exhibited the usual change of colour, from a green to a reddish hue.

*Observation* 6. In another fœtal calf of considerable size, (presumed to be in the ninth month of uterine existence, and which was examined twelve hours after death,) the stomach contained four ounces of a yellow glairy fluid, in which, as it lay in the stomachic cavity, were suspended three or four fibrinous masses of a dark, brown colour. On allowing these matters to stand for some hours in a cylindrical glass vessel, the quantity of the coagulum or sediment was very much increased, so that its bulk ultimately became equal to the tenth part of that of the supernatant liquid. This latter was slightly opaque from the presence in it of innumerable minute globules, resembling those formed during the coagulation of albumen, and though extremely viscid, it furnished no coagulum on the application of heat and nitric acid singly and in conjunction, the only effect produced being the gradual formation of a pellicle on its surface as the liquor evaporated. Of a few other tests which were applied, sulphuric and muriatic acids caused no change whatever in the appearance of the fluid ; ferro-cyanide of potassium, assisted with acetic acid, tincture of galls, sulphate of copper and alum, slightly increased its opacity, and the addition of a drop of solution of acetate of lead or proto-nitrate of mercury, was instantly followed by the appearance of an opaque membranous coagulum.

Imbedded in the coagulum or sediment, which was of a reddish brown colour and semi-transparent, were several thin yellow scales, perfectly opaque, and insoluble in boiling liquor potassæ, whereas the remainder of the fibrinous mass readily dissolved in that liquid. From the clear solution thus obtained, copious flakes were produced on its neutralization by muriatic acid.

In the mouth of this animal was found some glairy fluid, very similar to that met with in the stomach; and lying on the outer surface of the gums, particularly those of the lower jaw, were several of the peculiar yellow flakes just described.

The liquor amnii was clear, almost colourless, and, though somewhat viscid, did not with the usual tests afford any indication of albumen, being coagulated only by acetate of lead and proto-nitrate of mercury. *It contained none of the matters which constituted the coagula found in the stomachic fluid.**

*Observation* 7. The stomachs of two newly born kittens contained a brown, semi-transparent, viscid substance, which, on the application of heat or nitric acid, evinced the presence in it of a considerable quantity of albumen, but did not wholly coagulate. The portion which retained its transparency did not, however, manifest any disposition to dissolve in the boiling water. The contents of the small intestine were also albuminous.

In the œsophagus and fauces of one of these animals was found some of the same brown, viscid, semi-albuminous substances; and it may also be worthy of mention, that the quantity present in the stomach of this animal (which had lived a few hours after birth) was much less than in the other case.

*Observation* 8.—In each of two puppies, drowned immediately after birth, the stomach contained about half an ounce of a transparent, slightly viscid fluid, suspended in which were several large flakes of a light yellow or lemon colour, each flake being surrounded by an extremely tenacious gelatinous substance. On testing the clear supernatant liquid, it was found to be distinctly albuminous, an opaque stratum of coagula being deposited on the application of heat and nitric acid. A portion of one of the flakes and the adjacent mucus being examined under a high magnifying power, presented, in addition to epithelial scales and some oil globules, several minute particles of different shapes, a few appearing as perfect circles or rings, others of a crescentic form, and the remainder as rhomboidal and linear particles.

*Observation* 9.—The stomach of a human fœtus of about the fifth month, examined thirty hours after birth, was found moderately distended. On laying it open, a reddish coloured fluid escaped, and this was followed by three or four semi-transparent gelatinous masses. The largest of these was then placed in a watch-glass containing distilled water, and heat applied. As the temperature of the water approached the boiling point, numerous flakes were seen to radiate from the floating body; and as the process of ebullition proceeded, this latter became throughout its whole substance, firm and opaque, so as to constitute a perfect coagulum. Another mass was treated with nitric acid, and wherever the latter came in contact with it, an instantaneous coagulation took place. The contents of the small intestines were

* A quantity of the peculiar milky fluid, secreted by the spongy structures which receive the highly vascular fœtal cotyledons, and collected from them by pressure, on being heated, instantly coagulated into a firm mass. With the single exception of its colour being rather that of cream, than a distinct yellow, this substance precisely resembled the coagulum obtained by heating the contents of the stomach, of the mature fœtal rabbit. And in its chemical and nutritive properties this secretion presents a striking analogy to the colostrum furnished to the animal immediately after birth. The secretion of these structures was, in the mature fœtal lamb, also found to possess a similar property.

arranged in separate portions, in the order of their proximity to the stomach; and the liquid pervading them, on the addition of the same test, also evinced the presence of albumen, the relative quantity of this principle gradually decreasing as the chymous mass assumed the properties of meconium. My friend Dr. Glover was present at this observation, and kindly assisted me in its performance.

*Observation* 10.—The stomach of an infant, supposed to have died immediately after birth, but which was not examined till about forty hours afterwards, contained half an ounce of a remarkably viscid, reddish, semifluid substance, for the most part transparent, but presenting in its interior and on its surface, several opaque white striæ. On boiling a portion in distilled water, it became uniformly opaque, but did not acquire much additional firmness. Heated with strong nitric acid, a clear solution was obtained, from which, on its neutralization by liquor ammoniæ, a copious flaky deposit took place; and the same circumstance occurred with an alkaline solution, when rendered neutral by the addition of nitric acid. The white striæ were of a fatty nature, as evinced both by their chemical properties and microscopical appearance, being chiefly constituted of fat cells. A transparent viscid substance contained epithelial scales, oval nucleated cells, and a diffused granular matter. This substance could be traced into the duodenum, where it gradually became more opaque, and evidently constituted the material for chymification.

From all these facts, we may, I think, draw the following general conclusions:—

1. That the stomach of the fœtus, during the latter period of its uterine existence, invariably contains a peculiar substance, differing from the liquor amnii, and generally of a nutritious nature.

2. That in its physical and chemical properties, this substance varies very much in different animals, being in no two species precisely similar.

3. That in each fœtal animal the contents of the stomach vary much at different periods; in the earlier stages of its development, consisting chiefly of liquor amnii, to which the other peculiar matters are gradually added.

4. That the liquor amnii continues to be swallowed by the fœtus up to the time of birth; and consequently after the formation of those matters, and their appearance in the stomach.

5. That the mixture of this more solid and nutritious substance with the liquor amnii constitutes the material submitted to the process of chymification in the fœtal intestines.

The opinion that the fœtal chyle is principally formed from these matters, however, by no means implies a supposition that the *nutrition* of the fœtus is thus accomplished. For the actions which proceed in the chylopoietic viscera of the fœtus are, I imagine, chiefly useful from their gradually preparing the different organs for the important functions assigned to them in the economy of the mature animal. Whereas the nutrition of the fœtus is undoubtedly effected by its placental vessels, the venous capillaries of which (or to speak more correctly, the streams traversing them) possess an absorbing power precisely similar to that of the mesenteric veins. And as a portion of the fœtal blood, charged with the albuminous substance there absorbed, after passing through the umbilical vein, circulates in the blood-vessels of the liver, the presence of an albuminous fluid in the hepatic duct, as noticed by Drs. Lee and Prout, is by no means inexplicable. Viewed in this light, the umbilical vein of the fœtus will answer to the mesenteric veins of the adult, while the ramifications of the hepatic duct, in addition to their ordinary functions, may be considered as to a certain extent representing the lacteals.

The source of the peculiar substances found in the stomach of the fœtus still, however, remains to be determined. That they are not secreted by the stomach itself is, I think, rendered almost certain, by the uniformly pale, undeveloped condition of that viscus during fœtal life, and by the circumstance of their being occasionally met with in the fauces and mouth of the animal, as in observations 1, 6, and 7. And coupling this latter fact with the negative argument constituted by the impossibility of assigning their productions to any other organs, I am disposed to regard them as the secretion of *the salivary glands*, between the development of which and the gradual formation of these matters a certain degree of connection has appeared to me to exist.

Having thus briefly mentioned the facts met with in the course of this investigation, and indicated the conclusions which appeared to me to flow most directly from the consideration of those phenomena, I shall for the present abstain from any further inquiries into this department of physiology. But, before concluding this communication, I may be permitted to relate one or two additional observations, which tend to establish an interesting and important relation between two of the chief functions in the animal economy, viz. respiration and digestion.

I have before had occasion to corroborate, from my own observation, the statements of other inquirers as to the slight vascularity and torpid condition of the stomach during fœtal life. It became, therefore, an interesting problem to trace the gradual development of its digestive power, and to ascertain the precise period at which the effect of this power became perceptible.

The contents of the fœtal stomach were, in several of the foregoing observations, tested by litmus paper with very different results, the gastric fluid being in some cases neutral, sometimes alkaline, and in a few instances feebly acid. As the tissues and fluids of the fœtus, however, always manifest a strong tendency to acescent decomposition, and as the indication of acidity was generally noticed in cases where some delay had taken place in the examination of the gastric contents, I am not prepared to deny the possibility of this acescency having been the result of chemical changes taking place after death. A far more satisfactory test of the presence of the proper gastric juice, and consequently of the commencement of its digestive function by the stomach, was, I thought, to be found in the chemical action of that secretion upon albuminous fluids. And, as a liquid readily coagulating upon the application of all the common tests for albumen exists naturally in the stomach of the mature fœtal rabbit, a few observations upon these animals, performed at different periods after birth, promised to throw some light upon the subject.

*Observation* 11.—Two rabbits from the same litter were examined at the end of thirty hours after birth. In one (which from the appearance of the lungs, had evidently respired, but which was found dead within a few hours after the presumed time of birth,) the stomach, both as regards its vascularity and the nature of its contents, precisely resembled that of the advanced fœtus of the same species. Its coats were pale, and the blood-vessels distributed through them scarcely perceptible; while the substance within exhibited all its usual properties, being the same green, transparent, viscid, semi-fluid, coagulable mass as that invariably met with in the mature fœtus.

But the stomach and its contents in the other rabbit (which, though separated from the doe, had lived up to the moment of examination) presented a very different appearance. Its coats were beautifully injected with innumerable blood-vessels, and their thickness was apparently increased; whilst in its interior was *a large flattened coagulum of a greenish-yellow colour*, possessed of considerable tenacity, and exhibiting in its substance, when viewed microscopically, several oil globules, similar to those noticed in the uncoagulated matter.

Nothing could be more striking than the difference between these two stomachs; and, from a single positive observation like this, we are, I think, justified in concluding, that the formation of the gastric juice does not take place till the act of respiration has proceeded for a certain length of time, and rendered the oxygenation of the blood tolerably complete. In two other rabbits, killed at the end of twenty-four hours after birth, the albuminous contents of the stomach were similarly coagulated, its coats being also highly vascular, and a portion of the same green coagula being found in the duodenum. All these animals were removed from the doe before any milk could have been swallowed. In the following observation the young animal was allowed to feed with the rest.

*Observation* 12.—A young rabbit being killed the third day after birth, was instantly examined. The coats of the stomach, which was very much distended with food, were highly vascular, so as to assume a rosy tint. It contained a great mass of coagulated milk, in the midst of which, and rendered conspicuous by the snow-white ground on which they were placed, appeared two half-digested coagula, of a dark greenish-brown colour. These were evidently the remains of the fœtal secretion; and, in the duodenum of the same animal, at the distance of two inches from the stomach, were several small acicular particles of a fatty nature.—*London and Ed. Monthly Journ. Med. Science.*

